# Supplementary material for: Shorter Survival after Liver Pedicle Clamping in Patients Undergoing Liver Resection for Hepatocellular Carcinoma Revealed by a Systematic Review and Meta-Analysis
Source: Cancers (Basel). 2021 Feb 5;13(4):637. doi: 10.3390/cancers13040637 (PMC7916026; doi:10.3390/cancers13040637)
Supplement: Supplementary file 1 [file cancers-13-00637-s001.pdf]

cancers

MDPI

Supplementary Material

Shorter Survival after Liver Pedicle Clamping in Patients Undergoing Liver Resection for Hepatocellular Carcinoma Revealed by a Systematic Review and Meta-Analysis

Charles-Henri Wassmer, Beat Moeckli, Thierry Berney, Christian Toso and Lorenzo A. Orci

Table S1: Meta-analysis comparing baseline characteristics of the study groups.

	Number of Studies	Measure of Effect	Pooled Estimate	95% Confidence Interval	*p* Value
Gender (M : F)	11	Odds ratio	0.99	0.89 to 1.11	0.961
Underlying liver cirrhosis	11	Odds ratio	1.89	0.81 to 4.38	0.139
Alpha-feto protein (U/L)	5	Weighted mean difference	411.59 [U/L]	-965.31 to 1788.5	0.558
Tumor size (cm)	7	Weighted mean difference	0.10 [cm]	-0.366 to 0.568	0.673
Multinodular hepatocellular carcinoma	11	Odds ratio	1.02	0.88 to 1.19	0.812
